# Hepatitis-D Virus Infection Is Not Impaired by Innate Immunity but Increases Cytotoxic T-Cell Activity

**DOI:** 10.3390/cells10113253

**Published:** 2021-11-20

**Authors:** Sebastian Maximilian Altstetter, Oliver Quitt, Francesca Pinci, Veit Hornung, Aaron Michael Lucko, Karin Wisskirchen, Stephanie Jung, Ulrike Protzer

**Affiliations:** 1 Institute of Virology, School of Medicine, Helmholtz Zentrum München/Technical University of Munich, 81675 Munich, Germany; sebastian.altstetter@tum.de (S.M.A.); oliver.quitt@tum.de (O.Q.); aaron.lucko@tum.de (A.M.L.); karin.wisskirchen@tum.de (K.W.); 2 Gene Center and Department of Biochemistry, Ludwig-Maximilians—University Munich, 81377 Munich, Germany; pinci@genzentrum.lmu.de (F.P.); hornung@genzentrum.lmu.de (V.H.); 3 Institute of Cardiovascular Immunology, University Hospital Bonn, University of Bonn, 53127 Bonn, Germany; 4 German Center for Infection Research (DZIF), Munich Partner Site, 81675 Munich, Germany

**Keywords:** hepatitis delta virus, hepatitis B virus, innate immunity, MDA5, antiviral response, T-cell dependent cytotoxicity, T-cell engineering

## Abstract

Approximately 70 million humans worldwide are affected by chronic hepatitis D, which rapidly leads to liver cirrhosis and hepatocellular carcinoma due to chronic inflammation. The triggers and consequences of this chronic inflammation, induced by co-infection with the hepatitis D virus (HDV) and the hepatitis B virus (HBV), are poorly understood. Using CRISPR technology, we characterized the recognition of HDV mono- and co-infection by intracellular innate immunity and determined its influence on the viral life cycle and effector T-cell responses using different HBV and HDV permissive hepatoma cell lines. We showed that HDV infection is detected by MDA5 and -after a lag phase -induces a profound type I interferon response in the infected cells. The type I interferon response, however, was not able to suppress HDV replication or spread, thus providing a persistent trigger. Using engineered T-cells directed against the envelope proteins commonly used by HBV and HDV, we found that HDV immune recognition enhanced T-cell cytotoxicity. Interestingly, the T-cell effector function was enhanced independently of antigen presentation. These findings help to explain immune mediated tissue damage in chronic hepatitis D patients and indicate that combining innate triggers with T-cell activating therapies might allow for a curative approach.

## 1. Introduction

Hepatitis D virus (HDV) infection is a serious health problem and affects approximately 70 million people worldwide. Chronically infected patients live at increased risk of developing liver cirrhosis, hepatocellular carcinoma, or acute fulminant hepatitis as the most severe form of viral hepatitis. Treatment options are limited to interferon alpha (IFNα), which per se has low success rates, and inhibition of virus entry using bulvertide, a peptide derived from the N-terminus of the hepatitis B virus (HBV) large envelope protein used by HBV and HDV recently approved by the European Medicines Agency [1,2,3]. Consequently, further research on the special characteristics of HDV is urgently needed to identify new therapeutic options.

HDV is the only known human pathogenic satellite virus and does not encode for its own envelope proteins [4]. Since HDV requires HBV envelope protein expression for release and productive infection, it only occurs together with HBV as a helper virus under natural conditions [5]. HBV behaves like a “stealth” virus and is not sensed by nor actively interferes with the intrinsic innate immunity of the infected hepatocytes [6,7]. In contrast, HBV-HDV co-infection leads to a strong interferon induction [8]. In addition, macrophages are capable of sensing HBV triggering a proinflammatory cytokine response [6,7].

The innate immune system detects cellular damage and infections by recognizing pathogen-associated molecular patterns (PAMPs) that are characteristic of distinct groups of pathogens [9]. This immunorecognition is enabled by pattern recognition receptors (PRRs) of the innate immune system. A specific class of PRRs are Retinoic Acid Inducible Gene 1 (RIG-I)-like receptors (RLRs), which detect cytoplasmic double-stranded RNA as a hallmark of viral replication. This family includes RIG-I and Melanoma Differentiation Associated Gene 5 (MDA5) as activating receptors, as well as Laboratory of Genetics and Physiology 2 (LGP2) as an accessory molecule [10]. While RIG-I has been reported to recognize shorter double-stranded RNA with a 5′ di- or triphosphate modification, MDA5 was shown to recognize longer, double-stranded RNA and more complex RNA structures [11,12,13,14]. Activation of RLRs by their specific RNA PAMPs leads to intramolecular conformational changes, which enables their interaction with Mitochondrial Antiviral Signalling (MAVS) protein [15]. MAVS functions as a scaffold for subsequent signalling cascades which induce IFN production leading to the upregulation of interferon-stimulated genes (ISGs). Though MDA5 has recently been shown to be the HDV detecting receptor, the exact mechanisms of pattern recognition in HDV infection remain poorly characterized, as model systems have only recently become available [8,16,17].

We used permissive human cell lines to characterize HDV-triggered pattern recognition and to study the effects of innate immunity on HDV infection and HBV-HDV co-infection as well as on effector T-cell immunity. We found that innate immune sensing exclusively depended on MDA5 expression, but did not affect viral replication or the number of virus-infected cells. However, innate sensing of HDV PAMPs was correlated with enhanced T-cell dependent cytotoxicity in HBV-HDV co-infection.

## 2. Materials and Methods

### 2.1. Antibodies, Reagents and Kits

Cellular RNA from cell cultures was extracted using the NucleoSpin RNA isolation Kit (Macherey-Nagel, Düren, Germany) according to manufacturer’s instructions. For cDNA transcription from extracted cellular RNA, the SuperScript^®^ III First-Strand Synthesis System for RT-PCR kit was used according to the manufactures protocol. For ELISA, the Human IFN Beta ELISA Kit, High Sensitivity (Serum, Plasma, TCM) was used according to the manufactures protocol. HBV was produced as described and purification was done via heparin binding columns followed by caesium chloride gradient centrifugation [18].

### 2.2. AAV-HDV Production

HDV genome containing AAV vector production was based on transient transfections and performed as described [17]. Cells were harvested by pelleting at 1000 g for 15 min 72 h after transfection. Cells were then washed with PBS and resuspended in 7.4 mL AAV lysis buffer (50 mM Tris, 150 mM NaCl, 5 mM MgCl_2_ in H_2_O). Cell lysate was exposed to three freeze–thaw cycles and treated with 50 U/mL benzonase for 30 min at 37 °C. Purification of the AAV-HDV stock was performed via an iodixanol gradient ranging from 60% to 15% iodixanol. Centrifugation was carried out in a SW55Ti rotor (Beckmann Coulter, Brea, CA, USA) for 2 h at 50,000 rpm at 4 °C. After centrifugation, the AAV-HDV vector was collected from the 40% iodixanol phase. Viral DNA was isolated by incubating 5 µL virus stock with 5 µL TE Buffer (from the Plasmid Gigaprep Kit) and 10 µL NaOH (2M) for 30 min at 56 °C and adding 480 µL HCl (40 mM) thereafter. Viral genome titers were determined by qPCR using the AAVpro^®^ Titration Kit (Takara Bio, Kusatsu, Japan) following manufacturer’s instructions. AAV stocks were stored at −80 °C.

### 2.3. Cells

Plates and flasks were collagenized prior to cell seeding. NTCP-expressing HepG2-cells and RLR-expressing Huh7.5 cells were cultivated in D-MEM (Pan-Biotech, Aidenbach, Germany) supplied with 10% FCS, 2 mM L-Glutamine, 100 Units/mL Penicillin, 10 µg/mL Streptomycin, amino acids, sodium pyruvate and 30 µg/mL blasticidin as a selection marker. Prior to infection, cells were differentiated for 14 days by adding 2.5% DMSO (Sigma, St. Louis, MO, USA) to the growth medium. HepaRG-cells were cultivated in Williams medium (Invitrogen, Carlsbad, CA, USA) supplied with 10% non-heat-inactivated FCS Fetalclone II (HyClone, Thermo Scientific, Waltham, MA, USA) 2 mM L-Glutamine, 100 Units/mL Penicillin, 10 µg/mL Streptomycin, 0.023 IE/mL human insulin, 4.7 µg/mL hydrocortisone and 80 µg/mL Gentamicin. Cells were differentiated in 1.8% DMSO for 14 days prior to infection. Prestimulation of HepG2-NTCP cells was achieved using Polyinosinic:polycytidylic acid (Poly I:C) at a final concentration of 100 ng/mL.

### 2.4. CRISPR/Cas9 Mediated KO Generation

To generate gene-deficient Na^+^-taurocholate cotransporting polypeptide expressing HepG2-cells (HepG2-NTCP), 1 × 10^6^ HepG2-NTCP cells were plated in a 6-well plate and transfected with 2.5 µg of U6-sgRNA, 2.5 µg of CMV-PuroR-T2A-Cas9 in combination with 10 µL of GeneJuice transfection reagent. Transfected cells were selected with puromycin (0.5 µg/mL) for 6 days and then plated under limiting dilution conditions. Four weeks later, single clones were picked and rearranged to allow further culture and deep sequencing analysis, as described previously [19]. Three clones per knockout with out-of-frame mutations in both alleles and with no wild-type reads were identified using Outknocker [20] and utilized for further experiments. Functional knock-out was confirmed (see Appendix A, Figure A2) and HepG2-NTCP MDA5 KO A4, HepG2-NTCP RIG-I KO G7 and HepG2-NTCP MAVS KO D4 clones were selected for further experiments.

### 2.5. HBV and HDV Infection

Hepatoma cell lines were infected with HBV and/or HDV as described [21]. If not indicated otherwise, a multiplicity of infection (MOI) of 20 viral particles (vp)/cell was applied in HDV infection. As indicated in the figure legends, an MOI of 20 or 100 DNA-containing, enveloped vp/cell was applied in HBV infection. Transduction with adenovirus-associated virus-packaged HDV (AAV-HDV) was performed using the same protocol and an MOI of 2 × 10^4^ vp/cell.

### 2.6. HDV Production

HDV production was based on transient transfection of Huh7 cells with HDV-encoding pSVL(D3) [22] and HBV-surface protein encoding pT7HB2.7 [23]. FuGENE^®^ HD (Promega, Madison, WI, USA) was used as transfection reagent. HDV containing supernatants were collected for two weeks and purified using HiTrap Heparin HP affinity columns (GE Healthcare, Chalfont St Giles, UK). Viral particles in eluted fractions were concentrated using Vivaspin^®^ Turbo 15 columns (MWCO 50 kDa) (Sartorius, Göttingen, Germany). Concentration of viral genome equivalents was determined by qRT-PCR.

### 2.7. qPCR for HDV Detection

HDV genomes were quantified as vGE using QuantiTect Virus Kit (Qiagen, Hilden, Germany) in a one-step qPCR. Reverse transcription was performed for 20 min at 50 °C followed by an initial denaturation step (5 min at 95 °C). Amplification occurred in 45 cycles of sequential denaturation (15 s at 95 °C) and primer annealing and extension (45 s 60 °C) steps. Analysis was performed in the LightCycler 480 Real-time PCR 96-well system II (Roche, Basel, Switzerland). Following oligonucleotides were used: CCC TTA GCC ATC CGA GTG G (HDV fw), TCC TCT TCG GGT CGG CA (HDV rev), ATG CCC AGG TCG GAC CGC G (HDV probe). The 1st WHO International Standard for HDV RNA, genotype 1 (Cat. NO. 7657/12, provided by PEI) was used for quantification.

### 2.8. qPCR for Gene Expression Analysis

Total RNA (650 ng) isolated from hepatoma cells as described previously was reverse transcribed into cDNA using the Superscript III First-Strand Synthesis System (Invitrogen/Thermo Fisher Scientific, Waltham, MA, USA). Real-time qPCRs were performed with the LightCycler FastStart DNA MasterPLUS SYBR Green Kit using the LightCycler system and normalized to a dilution series of calibrator cDNA and expressed relative to reference gene TATA-binding protein 1 (TBP1) using the Relative Quantification Software (all Roche Diagnostics). Following oligonucleotides were used: ACT GTA CGC TGT ACC T (CXCL10 fw), TGG CCT TCG ATT CTG GA (CXCL10 rev), AGA GCT GGA CGG ATG TTA GC (OAS1 fw), GGT TTG GTG CCA GAA CTG AG (OAS1 rev), GAT CAG CCA TAT TTC ATT TTG AAT C (IFIT1 fw), GAA AAT TCT CTT CAG CTT TTC TGT G (IFIT1 rev), CTG CAG CAG TTC CAG AAG G (IFN-β fw), TCA TTC CAG CCA GTG CTC GA (IFN-β rev), ATT CCA GGT TGT CAT CAA TG (ADAR1 fw), GAT TCT TTC TCT GTG GAA TA (ADAR1 rev).

### 2.9. HDAg Immunofluorescence Staining and Analysis

To visualize HDV replication within infected cells, Hepatitis Delta antigen (HDAg) was stained intracellularly. Cells were seeded and differentiated on collagenized coverslips prior to infection and staining. Infected cells were fixed in 4% paraformaldehyde (PFA) for 15 min at room temperature. Cells were permeabilized in 0.25% Triton X-100 in PBS for 10 min. Non-specific antibody binding sites were blocked with 5% BSA in PBS for 1 h. HDAg staining was achieved with the primary antibody HDAg#280 (1:500 in 1% BSA) [24] for 1 h at room temperature. The secondary antibody was Alexa Flour 594 goat anti-mouse IgG (Jackson Immuno Research, West Grove, PA, USA) diluted 1:750 in 1% BSA and incubated with cells for 30 min at room temperature in the dark. After each step, cells were washed three times in 1 x PBS. After staining, coverslips were mounted with DAPI Fluoromount-G (SouthernBiotech, Birmingham, AL, USA) on a microscope slide. The slide was stored at 4 °C until analysis via the confocal microscope Fluoview FV10i (Olympus, Shinjuku, Japan) at 20 °C using acquisition software FV10i SW 02.01.01.07 was performed. Images were processed using software version FV10i ASW 04.02.03.02.

### 2.10. Realtime Cell Viability Assay with xCELLigence RTCA

An xCELLigence RTCA system (ACEA Biosciences, San Diego, CA, USA) was used to determine the impact of HDV innate immune recognition on cell viability. HepG2-NTCP cells were co-cultured with genetically modified HBV-specific T-cells [25,26] and T-cell induced antigen-specific killing rates were measured. Therefore, HepG2-NTCP and HepG2-NTCP MDA5 ko cells were differentiated for 14 days. Differentiated cells were co-infected with either HBV and HDV or only HBV and infection was established for seven days. Afterwards, infected cells were seeded at a density of 5 × 10^4^ cells/well on collagenized xCELLigence 96-well plates and rested for two more days prior to start of co-culture. T-cells were added to seeded cells in different effector (T-cell) to target (HepG2-NTCP cell) ratios (1:1, 1:3, 1:9). Cell viability was determined as cell index and normalized to the start of co-culture.

## 3. Results

### 3.1. HDV Infection Induces a Delayed Type I Interferon Response

**Figure 1:**
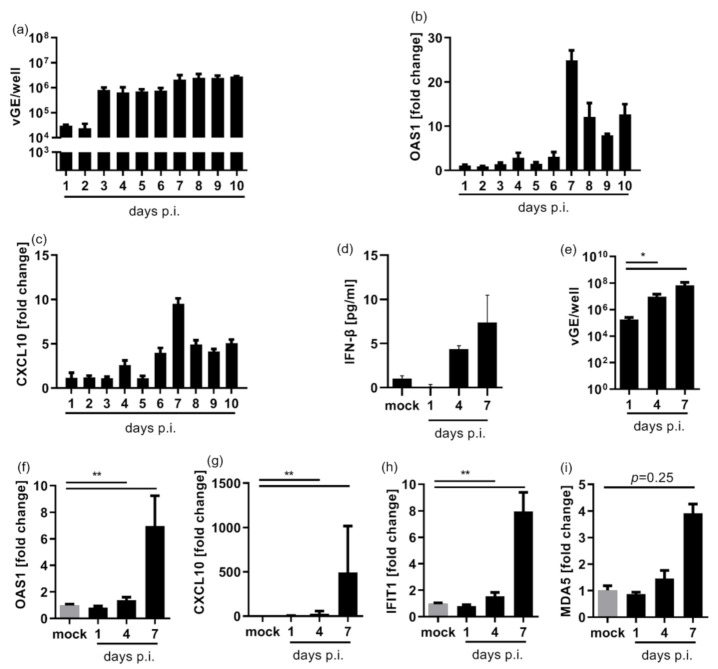
HDV replication induces a Type I interferon response in hepatoma cell lines. NTCP-expressing hepatoma HepaG2 and HepaRG cell lines were infected with HDV and extracted RNA was subjected to qRT-PCR. (**a**) Absolute numbers of vGE/well detected in HepaRG-cells seeded in a 6-well plate. Bars represent a single experiment with biological triplicates. (**b**,**c**) Upregulation of the ISG OAS1 (**b**) and CXCL10 (**c**) in HDV-infected HepaRG-cells is given as fold induction relative to non-infected cells. Graph depicts a single experiment with biological triplicates. (**d**) IFN-β release from HDV-infected HepG2-NTCP cells was determined by ELISA. Graph depicts a single experiment with biological duplicates. (**e**–**i**) HepG2-NTCP cells were seeded in a 24 well plate infected with HDV. Graphs depict absolute numbers of vGE/well (**e**) and upregulation of indicated ISGs OAS1, CXCL10,IFIT1 and MDA5 (**f**–**i**). (**e**–**h**) Mean ± SD of three independent experiments each in biological triplicates are given. Data were analysed for normality distribution using Kolmogorov–Smirnov test, statistical analysis of the normally distributed data was done using paired *t*-tests. * *p* < 0.05, ** *p* < 0.01 (**i**) Mean ± SD of one single experiment in biological triplicates is given. Data were analysed for normality distribution using Kolmogorov–Smirnov test, statistical analysis was done using Wilcoxon-test.

**Figure A1:**
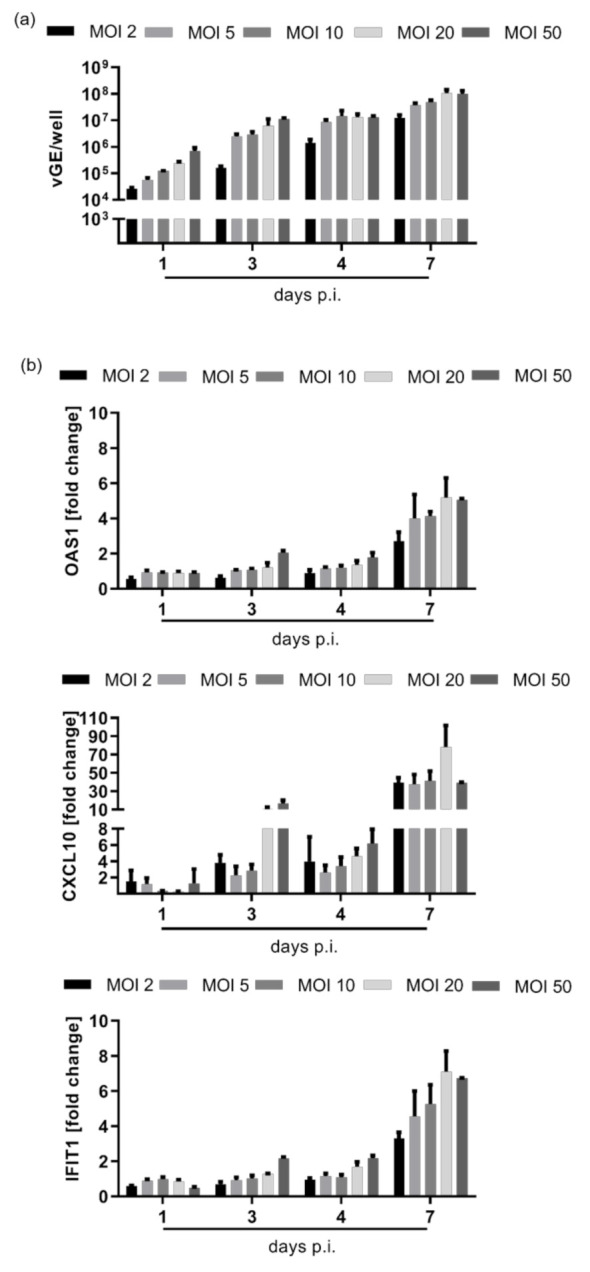
Dose-dependent increase in HDV replication and ISG expression. HepG2-NTCP cells were seeded in a 24-well plate, infected with HDV at the indicated MOI (vp/cell) and extracted RNA was subjected to qRT-PCR. (**a**) Absolute numbers of vGE/well detected in HepG2-NTCP cells. Bars represent one single experiment with biological triplicates. (**b**) ISG OAS1, IP10 and IFIT1 upregulation in HDV-infected HepG2-NTCP cells is given as fold induction relative to non-infected cells. Graph depicts a single experiment with biological triplicates.

To characterize the IFN response to HDV infection in two human hepatoma cell lines, we infected both HepaRG and HepG2-NTCP with HDV at an MOI of 20 vp/cell. Viral genome equivalents (vGE) showed a profound increase by 3 to 4 days post infection (dpi) (Figure 1a,e), indicating efficient viral replication. ISG expression, exemplified by Oligoadenylate-synthetase 1 (OAS1), C-X-C motif chemokine 10 (CXCL10), and Interferon Induced Protein With Tetratricopeptide Repeats 1 (IFIT1) upregulation as well as IFN-β release, increased after a lag phase on day 7 (Figure 1b–d,f–h). To further investigate the dependence of the IFN response on the dose of HDV genome equivalents, we infected HepG2 cells with different MOI’s of HDV particles/cell (Figure A1). A higher MOI enhanced the number of vGE produced and induced a stronger IFN response, but no statistically significant correlation between higher numbers of viral genomes and ISG expression levels was observed. However, independent of the infection dose, a profound increase in ISG expression occurred only on day 7. An IFN-dependent positive feedback loop, which increases MDA5-expression by a fourfold from day 1 to day 7, may be responsible for this effect (Figure 1i).

### 3.2. HDV Is Detected by MDA5

**Figure 2:**
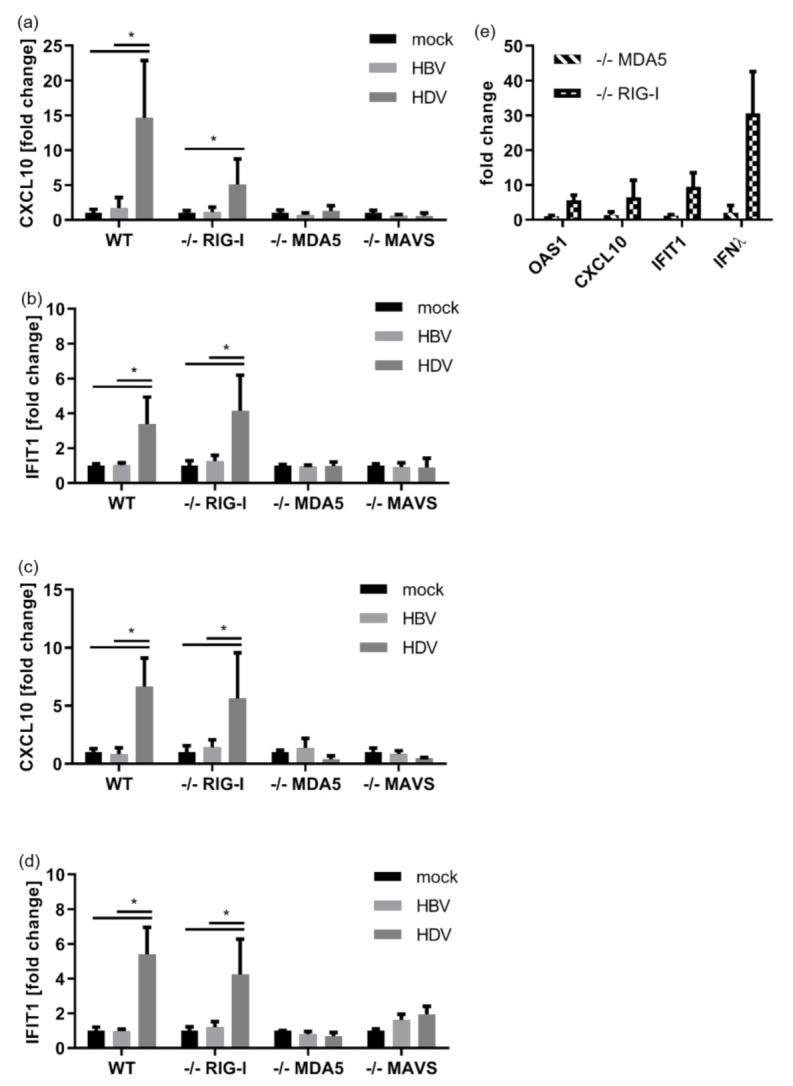
HDV is detected by MDA5. (**a**–**d**) HepG2-NTCP cells in which RIG-I, MDA5 or MAVS was knocked-out using CRIPSR/Cas9 were infected with HBV (light bars) or HDV (dark bars) at an MOI of 20 vp/cell each. RNA was extracted 7 (**a**,**b**) or 11 dpi (**c**,**d**) and subjected to qRT-PCR. Upregulation of (**a**,**c**) CXCL10 and (**b**,**d**) IFIT1 is given as fold induction relative to non-infected cells. Graphs depict a single experiment with biological quadruplicates. Data were analysed for normality distribution using Kolmogorov–Smirnov test, statistical analysis was done using Mann–Whitney-test. * *p* < 0.05 (**e**) MDA5-(striped bars) and Rig-I-knockout (plaid bars) HepG2-NTCP cells were transduced with AAV-HDV. RNA was extracted on day 11 dpi and subjected to qRT-PCR. Upregulation of the indicated ISGs and type III interferon λ (IFNλ) is given as fold induction relative to non-infected cells. Graph depicts a single experiment with biological duplicates.

**Figure A2:**
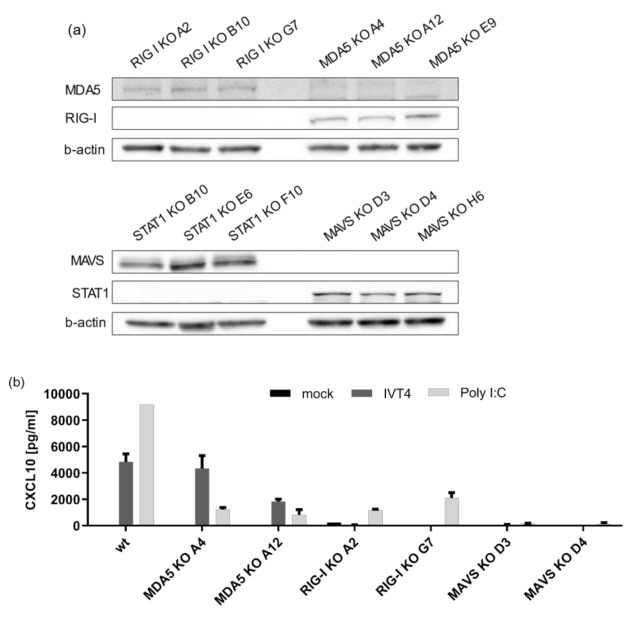
Western blot control of CRISPR-Knockout. HepG2-NTCP cells were subjected to CRISPR-knockout of RIG I, MDA5, MAVS and Signal transducer and activator of transcription 1 (STAT1, Western blot control). (**a**) Verification of MDA5, RIG I, MAVS and STAT1-knockout by Western blotting. MDA5-band is marked by arrow-head. Uncropped gels are shown. (**b**) Functional control of selected CRISPR-knockout cell lines upon stimulation experiment. Cells were stimulated as follows: 200 ng/well IVT4 (RIG I ligand) or transfected with 200 ng/well poly I:C, both RIG I and MDA5 ligand. Medium was collected 24 h post stimulation and CXCL10 ELISA was performed. Graph depicts a single experiment with technical duplicates.

**Figure A3:**
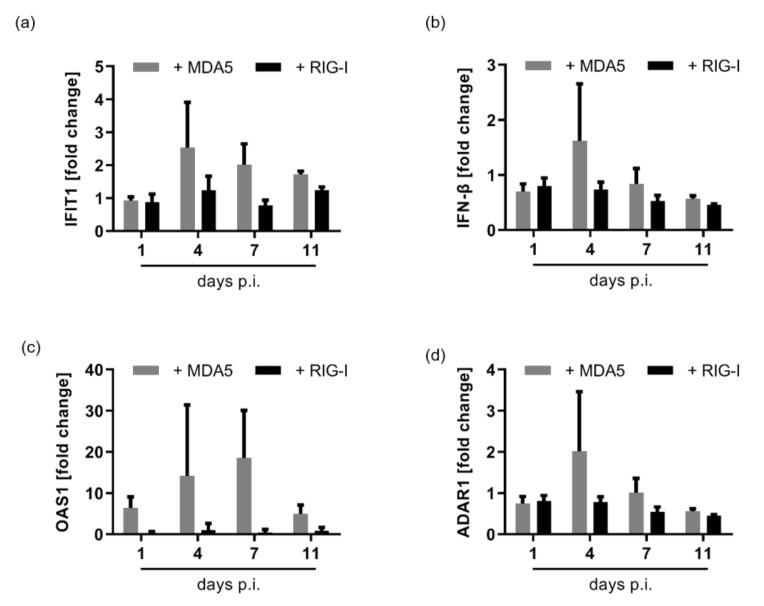
AAV-HDV infection upregulates ISGs in RLR-overexpressing Huh7.5 cells. Huh7.5 cells stably expressing MDA5 (+MDA5) or RIG-I (+RIG-I) were transduced with AAV-HDV. RNA was extracted at indicated days and subjected to qRT-PCR. Upregulation of the ISGs IFIT1 (**a**), IFN-β (**b**), OAS1 (**c**) and ADAR1 (**d**) is given as fold induction relative to cells not expressing RLRs at indicated timepoints. Graph depicts a single experiment in triplicates.

We next studied the involvement of different pattern recognition receptors in HDV recognition. We used a CRISPR/Cas9-mediated gene editing to generate HepG2-NTCP cells deficient for either RIG-I, MDA5, or MAVS protein. Knockout-efficiency and functionality of the remaining receptors in the selected monoclones were verified by Western blot and immune stimulation experiments using specific triggers (Figure A2). We confirmed that HDV immune recognition depends exclusively on MDA5 inducing MAVS signalling, since both WT and RIG-I deficient cells showed an IFN response to HDV infection, but not MDA5 and MAVS deficient cells (Figure 2a–d). To support this observation with a second experimental approach and to eliminate any potentially confounding effects of HBV proteins, we transduced RIG-I and MDA5-deficient HepG2-NTCP cells with an adenoassociated viral vector delivering an HDV genome (AAV-HDV). Again, the IFN response depended exclusively on MDA5 expression (Figure 2e). Additionally, we employed Huh7.5 cells which are naturally devoid of functional RLRs and stably transduced with MDA5 and RIG-I [27]. Delivery of HDV genomes by AAV-HDV resulted in MDA5-dependent ISG upregulation (Figure A3).

### 3.3. Intracellular Pattern Recognition of HDV Does Not Impair Virus Replication

**Figure 3:**
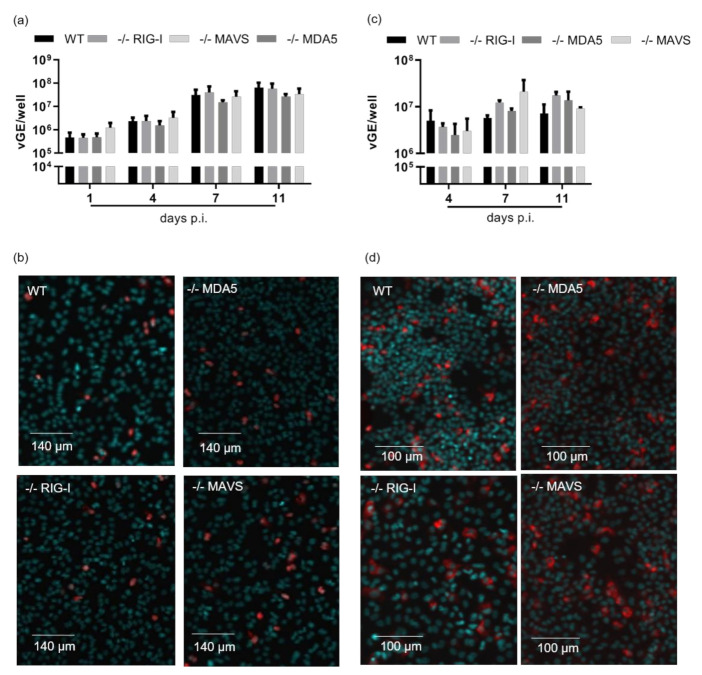
Intracellular pattern recognition of HDV does not impair virus replication. RIG-I, MAVS and MDA5 knockout-HepG2-NTCP cells were seeded in a 24-well plate, infected with HDV at an MOI of 20 vp/cell (**a**,**b**) or transduced with AAV-HDV at an MOI of 10^4^ vp/cell (**c**,**d**). (**a**) Absolute numbers of vGE/well detected in infected cells by qRT-PCR. Bars represent one single experiment with biological triplicates. (**b**) Exemplary HDAg immunofluorescence staining 11 dpi. Scale bars 140 µm. (**c**) Absolute numbers of vGE/well detected in infected cells by qRT-PCR. Bars represent one single experiment with biological triplicates. (**d**) Exemplary HDAg immunofluorescence staining 12 dpi. Scale bars 100 µm.

Having confirmed that HDV was sensed by MDA5 dependent on MAVS-signalling in HepG2-NTCP cells, we next investigated the effects of the interferon response induced on the viral life cycle. For this purpose, MDA5-/-, RIG-I-/- and MAVS-/- cells were infected with HDV and AAV-HDV. Surprisingly, both the increase in vGE (Figure 3a,c) and the number of HDV-expressing cells (Figure 3b,d) were completely independent of the presence of MDA5 or RIG-I and thus independent of the interferon response.

**Figure 4:**
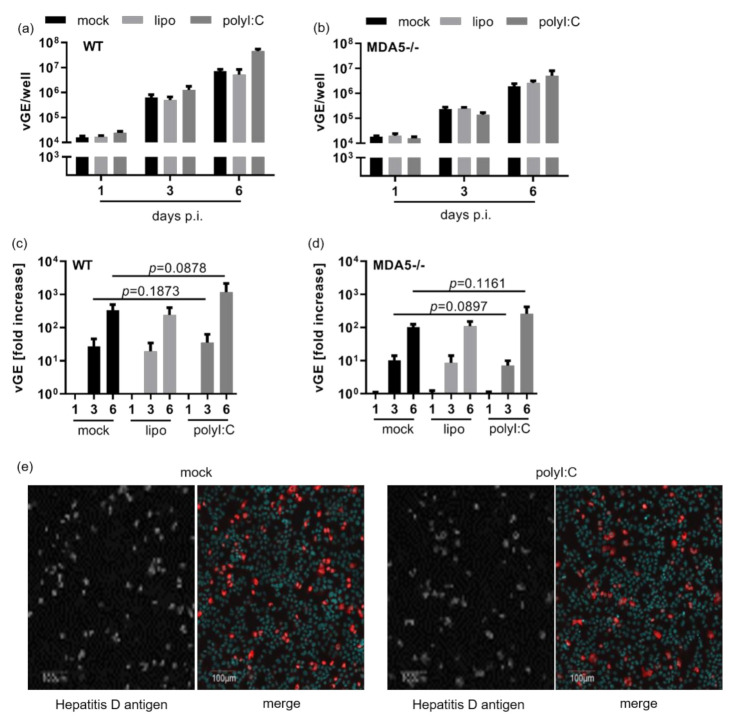
Type I interferon does not impair HDV replication. Both WT (**a**,**c**) and knock-out MDA5-/- (**b**,**d**) cells were seeded in a 24-well plate, stimulated with 100 ng/mL poly I:C using lipofectamine as control or left untreated (mock) and infected with HDV at an MOI of 20 vp/cell 6 h post treatment. RNA was extracted at 1, 3 and 6 dpi and vGE/well were quantified by qRT-PCR. (**a**,**b**) Absolute numbers of vGE/well detected in infected cells. Bars represent one single experiment with biological triplicates each. (**c**,**d**) Graphs depict the fold-increase in viral vGE over time relative to day 1. Bars comprise five data points per group from two independent experiments. Data were analysed for normality distribution using Kolmogorov–Smirnov test; statistical analysis of the normally distributed data was done using paired *t*-tests. (**e**) Exemplary HDAg immunofluorescence staining 7 dpi. Scale bars 100 µm.

**Figure A4:**
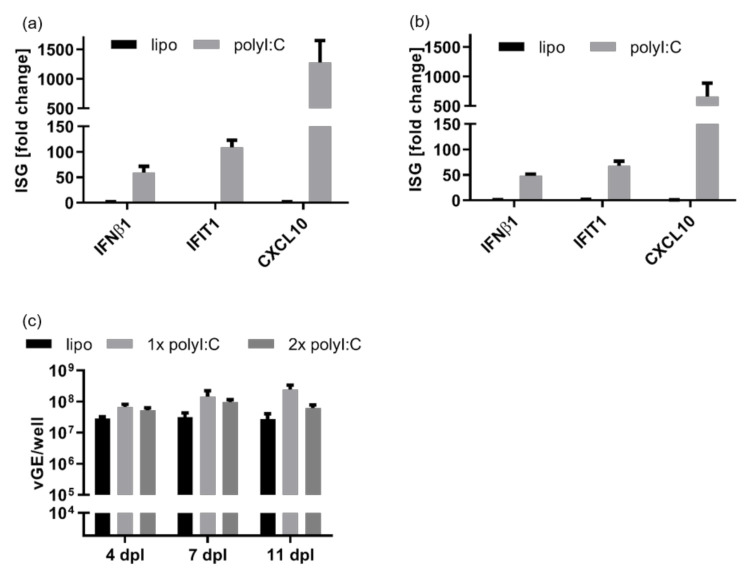
ISGs are specifically upregulated by poly I:C stimulation. Both WT (**a**) and MDA5-/- (**b**) HepG2-NTCP cells were stimulated with poly I:C at 100 ng/mL for 6 h. Extracted RNA was subjected to qRT-PCR and IFNβ and ISG upregulation is given as fold induction relative to non-stimulated cells. Graph depicts a single experiment with biological triplicates. (**c**) WT HepG2-NTCP cells seeded in a 6-well plate were stimulated with polyI:C at 100 ng/mL at 6 h pre-infection (1 x polyI:C) and re-stimulated with polyI:C at 100 ng/mL at day 4 post infection. Extracted RNA was subjected to qRT-PCR and absolute numbers of vGE/well are depicted. Bars represent a single experiment with biological triplicates.

Based on our observation in Figure 1 that ISG were only induced at 7 dpi, we suspected that the IFN response occurred too late to efficiently restrict HDV replication. To test this hypothesis, HepG2-NTCP cells were transfected with poly I:C before HDV infection. Lipofectamin only served as control. PolyI:C treatment led to a profound ISG induction prior to HDV infection (Figure A4a,b). However, neither the efficacy of HDV infection nor HDV replication were diminished by poly I:C pre-treatment and the interferon response induced, and there was no significant reduction of vGE over time (Figure A4a–d). Even repeated poly I:C treatment did not affect viral replication (Figure A4c). The number of HDV-expressing cells was also not affected by poly I:C pre-treatment (Figure 4e). Thus, we hypothesize that HDV infection itself—at least in HepG2 cells—is not sensitive to type I IFN.

### 3.4. Pattern Recognition of HDV Increases Cytotoxic T-Cell Killing of Infected Cells

**Figure 5:**
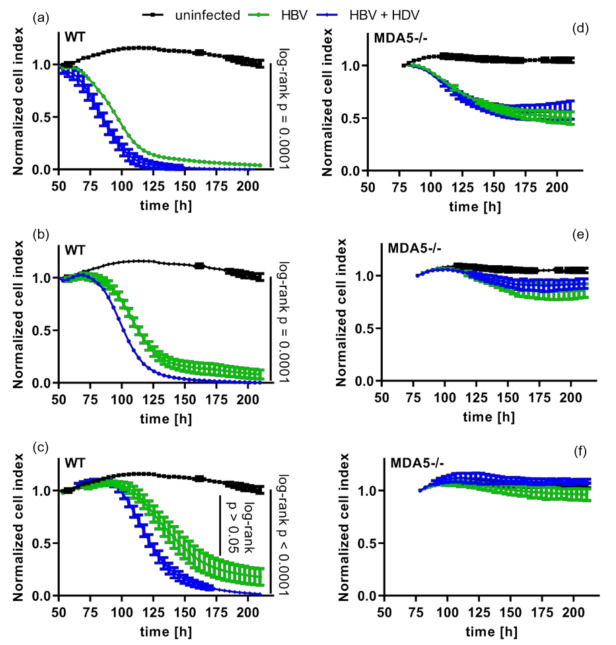
Pattern recognition of HDV sensitizes HBV-HDV coinfected cells to T-cell dependent cytotoxicity. Maternal and MDA5-/- HepG2-NTCP cells were infected with HBV at an MOI of 100 vp/cell and, if indicated (blue lines), coinfected with HDV at an MOI of 40 vp/cell. Cells were co-cultivated with 4G-TCR transduced T-cells at 9 dpi at an effector to target cell ratio of 1:1 (**a**,**d**), 1:3 (**b**,**e**) and 1:9 (**c**,**f**) and subjected to real-time cell viability assay. Graphs depict T-cell induced elimination of HBV infected cells reflected by a decreasing normalized cell index. Co-culture starts at 48 h. Graph represents a single experiment per line with biological triplicates. Statistical analysis of survival curves was done using Kaplan–Meier tests and log-rank analysis.

Since HDV, as a satellite virus, uses the HBV envelope and thus depends on HBV for a productive infection, we tested whether HDV immune recognition would influence the sensitivity to T-cell-mediated cytotoxicity. For this purpose, we used T-cells engineered to express the T-cell receptor (TCR) 4G recognizing a peptide derived from the HBsAg used by both viruses and presented on human leukocyte antigen HLA-A2 [26]. HepG2-NTCP cells coinfected with HBV and HDV were co-cultured with 4G-TCR-grafted T-cells (4G-TCR) after nine days of infection. We first confirmed that HBV monoinfection of WT cells facilitated 4G-TCR T-cell-dependent killing of HBV-infected cells, whereas uninfected cells were not affected (Figure 5a–c). Co-infection with HBV and HDV significantly increased this effect. However, TCR-dependent cytotoxicity was greatly diminished both in HBV monoinfected and in HBV-HDV coinfected MDA5-deficient cells, which could only be killed at high effector to target (E:T) ratios (>1:3) (Figure 5d–f). Most importantly, cytotoxicity was not increased further by HDV co-infection (Figure 5d–f).

**Figure 6:**
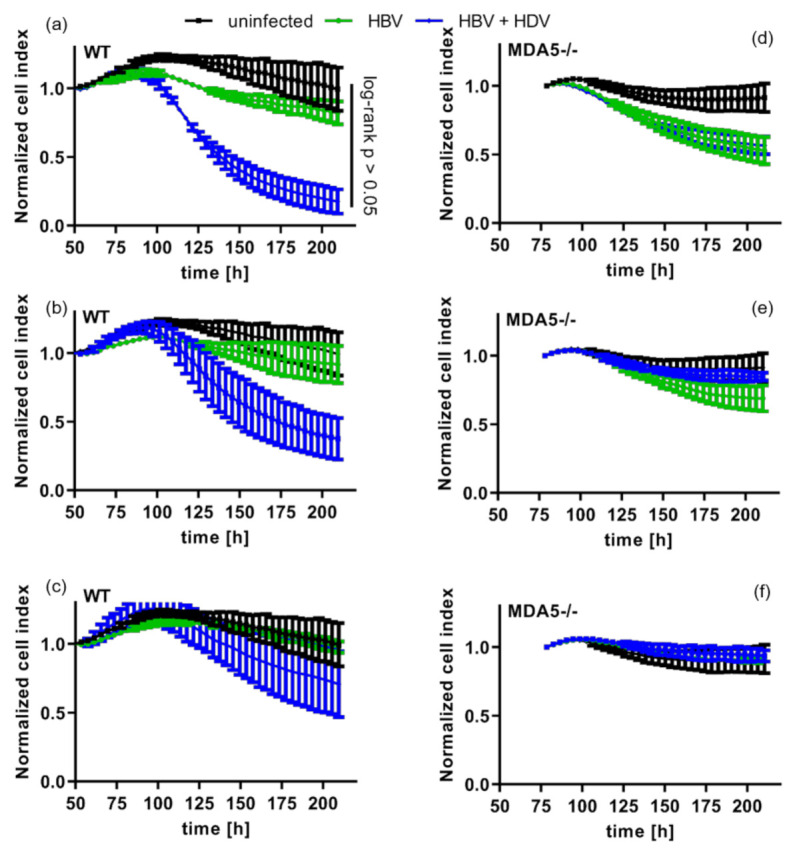
S-CAR T-cell dependent cytotoxicity independent of antigen presentation is increased by HDV recognition via MDA5. Maternal and MDA5-/- HepG2-NTCP cells were infected with HBV at an MOI of 100 vp/cell and, if indicated (blue lines), coinfected with HDV at an MOI of 40 vp/cell. Cells were co-cultivated with S-CAR transduced T-cells at 9 dpi at an effector to target cell ratio of 1:1 (**a**,**d**), 1:3 (**b**,**e**) and 1:9 (**c**,**f**) and subjected to real-time cell viability assay. Graphs depict T-cell induced elimination of HBV infected cells reflected by a decreasing normalized cell index. Co-Culture starts at 48 h. Graph represents a single experiment per line with biological triplicates. Statistical analysis of survival curves was done using Kaplan–Meier tests and log-rank analysis.

As MDA5-deficiency did not only diminish but also delay T-cell induced cytotoxicity by approximately 24 h (Figure 5d,e), we wondered whether the enhancement of T-cell-dependent cytotoxicity by HDV co-infection was caused by an increased antigen presentation due to innate immune activation. We therefore also used T-cells grafted with an HBsAg-specific chimeric antigen receptor (S-CAR) recognizing HBsAg on the surface of infected cells independent of antigen presentation on HLA [28] (Figure 6). Of note, HBV-HDV co-infection profoundly enhanced T-cell dependent cytotoxicity compared to HBV monoinfection when MDA5 was expressed (Figure 6a–c). MDA5 depletion abolished this effect (Figure 6d–f). Consequently, the increase in T-cell-dependent cytotoxicity by HDV co-infection did not depend on MHCI interactions but clearly depended on MDA5-mediated recognition of HDV.

In summary, HDV induces an MDA5-dependent IFN response, which is not able to suppress HDV replication. However, HDV immune detection leads to an increased sensitivity of infected cells to effector T-cell effector function and increases cytotoxicity.

## 4. Discussion

The high number of HBV-HDV co-infected patients, as well as the lack of curative therapies, underlines the need for a better understanding of the immunological processes involved in chronic viral hepatitis. How and when HDV infection is detected by the innate and adaptive immune systems is only partially understood, as the necessary model systems have only recently become available [8]. It is important to note that HDV is a satellite virus to HBV and that all studies should, therefore, include both HDV monoinfection and HBV/HDV co-infection. We established a series of HepG2-NTCP knockout cell lines in which HDV monoinfection as well as HBV/HDV co-infection induces a distinct innate immune response. These allowed to study the effects of innate and adaptive immunity upon mono- and co-infections.

We showed that HDV replicates in different hepatoma cell lines and induces a type I IFN response. Furthermore, we confirmed that HDV pattern recognition depends on MDA5 and MAVS-dependent signaling pathways in infected cells but also in cells transduced with an AAV-HDV. Interestingly, in all set-ups, a lag phase of several days was observed before ISGs were induced. Neither HDV-induced interferon nor activation of MDA5 and RIG-I by poly-I:C were able to inhibit HDV replication and spread. It, however, increased cytotoxicity of TCR and CAR-grafted T-cells, indicating that persistent HDV replication provides a persistent trigger of T-cell mediated, adaptive immunity.

While the number of vGE already strongly increased early after HDV infection, a significant induction of ISGs was observed 7 days after infection. This delay in the induction of innate immune response raises the question of whether a certain threshold of HDV replication related PAMPs must be exceeded before innate immune activation occurs or whether pattern recognition receptors must first be induced or pre-activated. However, this effect could not be circumvented by increasing the HDV infection dose, so a threshold for innate immune activation is unlikely. Nevertheless, the late induction of the innate immune response could be the reason why HDV-induced IFN signaling in mice and cell lines does not reduce viral replication [16,17].

While HBV is regarded a stealth virus, or fundamentally blocks the immune response [29,30,31,32,33,34,35,36,37], various publications report immune activation by HDV infection [8]. HDV was reported to induce both a type I and type III IFN response in the infected hepatocytes [16,17] and proinflammatory cytokine expression in neighbouring macrophages due to vesicular transfer of HDV-induced components [38]. In order to investigate the HDV-induced immune activation in more detail, we established various PRR-deficient cell lines by CRISPR/Cas9-mediated editing. Toll-Like Receptor 3 (TLR3), RIG-I and MDA5 can serve as HDV-detecting PRRs, as they all detect dsRNA and are functionally expressed in the liver [33,39,40,41]. While increased ISGs expression in response to HDV infection occurred in WT and RIG-I-ko cells, MAVS and MDA5 knockout completely abolished immune activation. This effect was also observed in cells transduced with AAV-HDV. Independent of the helper virus, HDV immune recognition depended exclusively on MDA5, but not on RIG-I or TLR3.

These results are consistent with previously published data [16,17], and provide an explanation why HDV is an HBV-associated satellite virus, although it has been shown that HDV may also spread with the help of other viruses [42]. Various publications report an inhibition of MAVS-dependent signalling by the HBV X protein [43,44,45,46,47], HBV polymerase [48] or HBV-induced microRNA [49], which in turn could impair the detection of HDV. However, it is still unclear how HDV RNA interacts with the cytoplasmic MDA5, since HDV replication has been shown to exclusively occur in the nucleus [50,51]. This compartmentalization, as well as the nucleoplasmic encapsidation of the HDV genome by the delta antigen, should theoretically lead to a spatial separation of HDV RNA from PRRs [9]. Furthermore, it is of particular interest how the MDA5-induced ISG response influences HDV replication.

Interestingly, HDV replication and spread was not affected by MDA5-dependent pattern recognition in either HDV or AAV-HDV infected cells. The number of infected cells in the culture was also independent of MDA5 expression. Consequently, we investigated whether HDV was generally insensitive to the interferon response, or whether this observation was caused by the delayed ISG expression. Pre-stimulation of the IFN response with Poly I:C also did not reduce viral replication or the number of infected cells. This was consistent with previously published studies, which demonstrate HDV-induced IFN-signalling in cell lines and mice without the reduction in viral replication [18,19,31]. It has also been reported that HDV can inhibit STAT-signalling [52] or may even benefit from type I IFN response and proinflammatory cytokine production [8]. In summary, HDV replication was not impaired by the induction of the innate immune response.

To further investigate why HBV/HDV co-infection causes such a severe liver inflammation, we investigated whether induction of the innate immunity upon HDV pattern recognition could affect adaptive T-cell responses. Since HDV only encodes for two proteins that largely overlap in their sequence, few antigens are accessible to MHC-dependent presentation and T-cell mediated immunity [8]. However, HDV depends on the expression of HBV envelope proteins for productive release and viral spread. Thus, HDV could affect HBV-specific T-cell function. Indeed, we showed that MDA5-dependent detection of HDV leads to enhanced HBV envelope protein specific T-cell cytotoxicity. These findings are consistent with studies of Tham et al., who reported that HBV-HDV co-infection led to an enhanced elimination of HBV-infected cells by cytotoxic T-cells [53]. As HBV-HDV co-infection, compared to HBV monoinfection, also leads to an upregulation of the IFN release, as well as all genes required for antigen processing and presentation, the authors suspected these gene products to be responsible for the enhanced elimination rate. However, as we observed the same effect using S-CAR T-cells acting independent of antigen presentation [28], we conclude that this effect does not depend on antigen presentation, but rather on IFN-mediated upregulation of cell death pathways like the Fas/Fas ligand pathway that could increase sensitivity towards T-cell killing [54]. It remains ambiguous why MDA5 deficiency also impaired and delayed T-cell dependent killing of HBV-monoinfected cells. HBV has been reported to induce type III IFN in a RIG-I-dependent manner [55], but no immunorecognition of HBV by MDA5 has been reported so far. One could thus speculate that HBV-RNA might be recognized by both RIG-I and MDA5, as these two evolutionary related receptors bind similar subsets of RNA ligands [56]. Alternatively, cellular RNA species have also been reported to be exposed upon viral infection, inducing RLR activation [57,58,59,60]. These RNA species might be induced by HBV infection itself, or by proliferation activity of HepG2-NTCP cells as a cancer derived cell line [59]. This way, a minor activation of the innate immune system and a subsequent upregulation of immune effector molecules via as yet unknown immunostimulatory RNA species could be responsible for enhanced T-cell dependent cytotoxicity.

Regardless of the exact mechanisms of action, our results should be further tested for their applicability in clinical settings. Presently, no cure for chronic HBV-HDV infection is available and patients require continuous treatment. IFN-α therapy as the only approved treatment option usually leads to low success rates [61]. Furthermore, unspecific therapies like Myrcludex B (Bulevirtide), the farnesyl transferase inhibitor (Lonafarnib), or nucleic acid polymers (REP 2139-Ca) are in phase II clinical trials [1]. Alternatively, elimination of HBsAg-positive liver cells by a specific T-cell response has shown promising results and grafting of HBV-specific T-cells has been shown to cure HBV-infected mice [25,26]. Our results demonstrate a clear effect of innate immune response on T-cell-mediated elimination of HBV-HDV coinfected hepatocytes. Further studies should clarify the exact mechanism of the MDA5-dependent increased sensitivity of HBV-HDV co-infected hepatocytes to cytotoxic T-cell responses.

In summary, we confirmed previous data that HDV is recognized by MDA5, which in turn induced an activation of innate immunity. Strikingly, this immune response is only indirectly able to restrict HDV infection by increasing the efficiency of T-cell mediated killing. Hereby, the essential helper virus HBV functions as the main target for adaptive immunity. The fact that specific elimination of HBV-HDV co-infected hepatocytes by HBV-specific T-cells was enhanced through immunorecognition of HDV infection opens the possibility to combine PRR stimulation with therapies like a therapeutic vaccine activating virus-specific T-cells.

## 5. Conclusions

We showed that HDV infection induces a specific, intracellular innate immune response which had no direct inhibitory effect on viral infection or replication. However, it promoted an active, cytotoxic T-cell response directed against the envelope protein shared by HDV and HBV, which serves as a helper virus and is therefore essential for HDV. In this way, persistent HDV replication and immune recognition can lead to a chronic, mainly T-cell mediated inflammation.

## Data Availability

Not applicable.
